# Impact of Nutrition Care Process Documentation in Obese Children and Adolescents with Metabolic Syndrome and/or Non-Alcoholic Fatty Liver Disease

**DOI:** 10.3390/healthcare9020188

**Published:** 2021-02-09

**Authors:** Gadah Mujlli, Dara Aldisi, Ghadeer S. Aljuraiban, Mahmoud M. A. Abulmeaty

**Affiliations:** 1 Clinical Nutrition Program, Community Health Department, King Saud University, Riyadh 11362, Saudi Arabia; Ghadah.a.almujlli@gmail.com (G.M.); daldisi@ksu.edu.sa (D.A.); galjuraiban@ksu.edu.sa (G.S.A.); 2 Education, Simulation and Skills Development Center, Princess Nourah Bint Abdulrahman University, Riyadh 11564, Saudi Arabia; 3 Obesity Management and Research Unit, Medical Physiology Department, Zagazig University, Zagazig 44519, Egypt

**Keywords:** nutrition care process, dietitian documentation, non-alcoholic fatty liver disease, metabolic syndrome

## Abstract

This study evaluated the Nutrition Care Process documentation used by dietitians for obese pediatric patients diagnosed with metabolic syndrome (MetS) and/or non-alcoholic fatty liver disease (NAFLD) and its impact on the achievement of nutritional goals. This retrospective cohort study utilized data retrieved from three tertiary care hospitals in Riyadh. A total of 142 obese pediatric patients aged 8–18 years diagnosed with NAFLD and/or MetS were evaluated. Data on weight, height, blood pressure (BP), lipid profile, and liver enzymes were collected. A validated audit was used to assess the documentation quality. Twenty-seven (46.6%) dietitian notes received a high score, 21 (36.2%) received a medium score, and 10 (17.2%) received a low score. There was no significant effect of dietitian audit scores on nutritional outcomes, however, the change in body mass index from 6 to 12 months follow-up period was inversely correlated with the audit score (*r* = −0.761, *p* = 0.007), and alkaline phosphatase was inversely correlated with the audit score (r = −0.819, *p* = 0.013). In conclusion, there was a clear variation in the quality of dietitians’ documentation and the impact of documentation scores on nutritional outcomes.

## 1. Introduction

Improving the quality of documentation in healthcare settings has been an ongoing process in various healthcare professions. This need arose from the increasing rate of preventable medical errors that result from poor quality documentation [1]. With the recent advancements in technology, electronic medical records have been implemented in the healthcare system, and their subsequent implementation has both reduced the incidence of adverse events resulting from medical errors and improved the quality of documentation [2]. In addition to patient safety, documentation quality plays an important role in the efficiency of work in an interdisciplinary healthcare team [3].

Dietitians, like other healthcare practitioners, are obligated to communicate with other members of the health team through documenting patient information, nutritional recommendations, and nutritional orders [4]. In 2013, the Academy of Nutrition and Dietetics established and implemented the standardized Nutrition Care Process (NCP) and Model [5]. The Academy of Nutrition and Dietetics (eatright.org) defines the NCP as “a systematic problem-solving method that dietetic professionals use to critically think and make decisions to address nutrition-related problems and provide safe and effective quality nutrition care” [5]. It consists of four interrelated concepts and steps: nutrition assessment, diagnosis, intervention, and monitoring and evaluation. The main purpose of this process is to give each dietitian a consistent and systematic structure that enables them to think critically and make decisions based on their conclusions. After developing the NCP, the Academy of Nutrition and Dietetics developed a new format for dietitian documentation to document the NCP steps; this new format is abbreviated ADIME [6].

A limited number of studies have focused on assessing the quality of dietitian documentation. Lövestam et al. performed a retrospective audit on 147 outpatient dietitian notes [7]. The notes were scored with a validated audit instrument (Diet-NCP-Audit) and divided into three different quality levels based on the total score: A (high score), B (medium score), or C (low score). Based on these levels, comparisons were made between the dietitians’ notes. The audit results indicated that most of the notes were classified as level B (score 13.5–19.5), and only 3% of the notes scored higher than 19.5. The most frequently documented items were nutritional interventions (90%) and evaluations (70%), while the least documented items were the goal of the intervention (9%) and the connection of problem etiology-symptoms (5%). Additionally, flaws in lingual clarity were commonly reported (72%) [7].

Metabolic syndrome (MetS) is a rising epidemic, with a worldwide prevalence ranging from 6.5% to 10%; this prevalence increases to 30% among overweight pediatric patients [8,9,10,11]. MetS has been considered a risk factor for type 2 diabetes mellitus (T2DM) and cardiovascular disease (CVD), and it has recently been associated with non-alcoholic fatty liver disease (NAFLD), the hepatic manifestation of MetS [12,13]. There are several risk factors associated with MetS, such as a sedentary lifestyle, high waist circumference, high body mass index (BMI), and a high-calorie diet [9]. Weight management and nutritional interventions comprise the first approach used for treating obesity, MetS, and NAFLD [14,15,16]. It is important to follow recent recommendations for implementing the NCP systematic approach to establish an individualized nutritional intervention for patients and to accurately document the process in the dietitian notes.

Limited studies in Saudi Arabia have aimed to evaluate dietitian documentation and its effect on nutritional outcomes in obese pediatric patients diagnosed with NAFLD and MetS comorbidities. Therefore, our study aimed to evaluate the NCP documentation in the medical records of Saudi hospitals for obese pediatric and adolescent patients diagnosed with MetS and/or NAFLD and to evaluate the impact of NCP documentation on the achievement of nutritional goals among obese pediatric and adolescent patients diagnosed with MetS and/or NAFLD.

## 2. Materials and Methods

### 2.1. Study Design and Setting

This retrospective cohort study utilized data retrieved from hospital medical records between 2014 and 2019. It was conducted in three tertiary care hospitals located in Riyadh, referred to as Hospital A, Hospital B, and Hospital C.

### 2.2. Study Participants

The study subjects included obese patients aged 8–18 years old who were already diagnosed with NAFLD and/or MetS. The inclusion criteria were as follows: male or female pediatric patients aged 8 to 18 who were diagnosed with obesity (Group I), obesity associated with NAFLD (Group II), obesity associated with MetS (Group III), or obesity associated with both NAFLD and MetS (Group IV). All participants may be firstly diagnosed with the above conditions at 8 years old or at an earlier age. NAFLD was diagnosed according to the definition of definitive NAFLD, which states that fat is present in the liver parenchyma. Ultrasound, CT, or liver biopsy and histology were used to diagnose NAFLD [17]. The subjects had specific parameters in their records to evaluate MetS within six months of NAFLD diagnosis (weight, height, blood pressure (BP), lipid profile, fasting blood glucose (FBG), or fasting insulin, liver function test (LFT)). The exclusion criteria included a history of parenteral nutrition, bariatric or hepatobiliary surgery, human immunodeficiency virus infection, metabolic acidosis, renal dysfunction, and short bowel syndrome.

### 2.3. Hospital Medical Record Search

This study was approved by Hospital B (IRP, E-19-3771) and Hospital A (IRP, H-01-R-005). Hospital C approved this study after reviewing the IRP registration memo submitted by the research team. After obtaining approval from the institutional review boards of the research ethics committees in the hospitals, the database of each hospital was searched for study subjects. The search for subjects differed in each hospital due to differences in the medical records systems. In Hospital A, the search was conducted using the following keywords: obesity, obese, non-alcoholic fatty liver disease, steatosis, and steatohepatitis; however, in Hospital C and Hospital B, searching was only possible through the radiology department using the following keywords: non-alcoholic fatty liver disease and non-alcoholic steatohepatitis. All cases that complied with the inclusion and exclusion criteria were included in this study.

### 2.4. Sample Size and Sampling Technique

This study required sample size of 200 obese patients with or without NAFLD and MetS complications. The sample size was calculated using Lenth, R. V (2006) Java Applets for Power and Sample Size [18]. This sample size was necessary to detect a difference in the proportion of 20% between groups with a power of 80%. A consecutive sampling technique was used to recruit the study subjects, and all patients who were eligible for the study were included in the data collection.

### 2.5. Study Parameters

#### 2.5.1. Socio-Economic Data and Medical History

Gender, age, and medical history were extracted from each medical record. The medical history included the patient’s diagnosis and current medications.

#### 2.5.2. Obesity Parameters

Weight and height were extracted from the medical records to calculate BMI. Additional information was extracted regarding how the patients were diagnosed with obesity, including growth charts, BMI, and/or waist circumference.

#### 2.5.3. Metabolic Syndrome Parameters

MetS was evaluated by the researcher based on the presence of at least three of the following criteria, as described by the Adult Treatment Panel III (ATP III) modified criteria for pediatrics [19]. This definition was used instead of the International Diabetes Federation (IDF) criteria because waist circumference, which is the criteria for defining obesity according to the IDF, is not measured in most hospitals in Riyadh.

Obesity: defined as a BMI ≥ 95th percentile according to the Center for Disease Control (CDC) growth charts. BMI was calculated using the following formula: body weight (kg)/height squared (m^2^). The degree of obesity was determined using the CDC BMI-age growth charts.

Abnormal glucose homeostasis: defined by the presence of one of either hyperinsulinemia and IR (using fasting blood insulin appropriate for the pubertal stage) or elevated fasting glucose (FG) ≥ 100 mg/dL.

Hypertension: defined by a systolic BP (SBP) or diastolic BP (DBP) ≥ 95th percentile for age and sex. BP was evaluated using “The fourth report on the diagnosis, evaluation, and treatment of high BP in children and adolescents” [20].

Dyslipidemia: defined by the presence of high triglyceride (TG) levels (≥95th percentile for age and sex), low high-density lipoprotein (HDL) levels (<5th percentile for age and sex), or high total cholesterol (TC) or low-density lipoprotein (LDL) cholesterol levels (≥95th percentile for age and sex). Dyslipidemia was defined using the subject’s lipid profile that included TG, TC, HDL, and LDL according to Hickman et al. [21].

#### 2.5.4. NAFLD Parameters

The following parameters related to NAFLD were recorded: NAFLD diagnosis method (either by abdominal imaging (ultrasound, computed tomography, magnetic resonance imaging) or liver biopsy and histology); diagnosis with ultrasound; stages of the disease (steatosis, non-alcoholic steatohepatitis, cirrhosis); and liver function test results including alanine aminotransferase (ALT), aspartate aminotransferase (AST), alkaline phosphatase (ALP), gamma-glutamyl transpeptidase (GGT), bilirubin, prothrombin time (PT), and C-reactive protein (CRP), which is an inflammatory marker. The severities of the NAFLD diagnostic parameters were collected from the records based on physicians’ reports and radiology reports.

When a subject is diagnosed via ultrasound, the stages of NAFLD are observed as follows: the mild stage, which displays a slight diffuse increase in the hepatic parenchyma, and normal visualization of the borders of the intrahepatic vessel and diaphragm; the moderate stage, which displays a moderate diffuse increase in fine echoes and slight impairment in the visualization of the intrahepatic vessels and the diaphragm; and the advanced stage, which displays a marked increase in fine echoes and poor to no visualization of the borders of the intrahepatic vessel, diaphragm, and the liver’s right lobe posterior portion [19,22]. When diagnosed via liver biopsy, the NAFLD activity score (NAS) is calculated based on the grades of steatosis (0–2), lobular inflammation (0–3), and ballooning degeneration (0–2). The total possible score ranges from 0 to 8. The stages of NAFLD are classified according to the NAS score: a score of 1–3 is stage 1, a score of 4–5 is stage 2, and a score of 6–8 is stage 3 [23].

#### 2.5.5. Assessing the Quality of Dietitians’ Documentation

Dietitian notes were viewed from each patient’s record and evaluated using Diet-NCP-Audit, a validated audit instrument, to assess the quality of dietitian documentation (Table 1) [24]. This tool was designed to assess the documentation of the four steps of the NCP, clarity of language, and structure of the notes. The tool contains 14 items, with 10 items that focus on the NCP steps and four items that focus on language clarity and structure. The first 12 items are scored from 0 to 2 depending on documentation quality, and items 13 and 14 are scored from 0 to 1, resulting in a total possible score of 26 points. The results are interpreted based on the total score as follows: high score, level A (20–26 points); medium score, level B (13.5–19.5 points); and low score, level C (0–13 points). This audit instrument was used for auditing dietitian documentation from the subject’s first visit because of the lack of research supporting the use of the tool to audit follow-up documentations.

#### 2.5.6. Achievement Parameters of Nutritional Goals

The parameters of the nutritional goals were recorded for each disease based on the subjects’ diagnoses. These parameters were collected at baseline and followed up after the dietitian’s first visit. If the subject was obese, weight management was the nutritional goal; thus, weight and height were recorded to calculate BMI. If the subject was diagnosed with NAFLD, weight reduction and improving the liver enzyme levels (ALT, AST, ALP, GGT, and bilirubin) were considered the nutritional goals; thus, weight and height were recorded to calculate BMI, and liver enzyme levels were determined. If the subject was diagnosed with MetS, the parameters of nutritional goals included: weight and height to calculate BMI, BP measurements, and lipid profile measurements including TG, TC, HDL, LDL, very-low-density lipoprotein (VLDL), fasting insulin, and FBG. The achievement of nutritional goals was indicated by improvement in the above-mentioned parameters. The subjects were assessed three times: first assessment (baseline), second assessment (six months after baseline), and third assessment (12 months after baseline). The disease parameter progression was computed using the following equation: change from baseline to 12 months = 1st assessment–3rd assessment.

### 2.6. Statistical Analysis

In this study, the independent variable was the score of the dietetic notes audit (continuous) which was also categorized according to the total score into three levels (categorical), and the dependent variables were the achievement of nutritional goals which were shown as changes of nutritional outcomes scores in study subjects (continuous variables). The data analysis was performed using the Statistical Package for Social Sciences version 25 (SPSS 25, IBM, Armonk, NY, USA). The progression of disease parameters was analyzed for the following groups: obesity (group I), obesity associated with NAFLD (group II), obesity associated with MetS (group III), and obesity-associated with both NAFLD and MetS (group IV). A paired T-test was used to evaluate the differences between the two assessment periods, and Freidman’s ANOVA was used to indicate the difference in the mean between variables collected during the three assessment periods. The audit tool evaluating the quality of dietitian documentation (quantitative-continuous) was categorized into three groups based on the total score: high, level A (20–26 points); medium, level B (13.5–19.5 points); and low, level C (0–13 points). The results are shown as descriptive statistics for each hospital. T-tests were performed to estimate the differences in disease parameters between the subjects with reported dietitian documentation and the subjects with no reported dietitian documentations. A sub-analysis was conducted using a repeated-measures ANOVA to estimate the effect of the level of dietitian documentation on the mean disease parameters. Spearman’s correlation analysis was conducted to assess the correlation between the achievement of nutritional goals and the dietitian documentation audit scores. A test with a *p*-value of < 0.05 was considered statistically significant, and correlations were considered strongly negative if the correlation coefficient *r* < −0.5 and strongly positive if *r* > 0.5.

## 3. Results

### 3.1. Study Subjects’ Demographic Data and Baseline Characteristics

**Figure 1:**
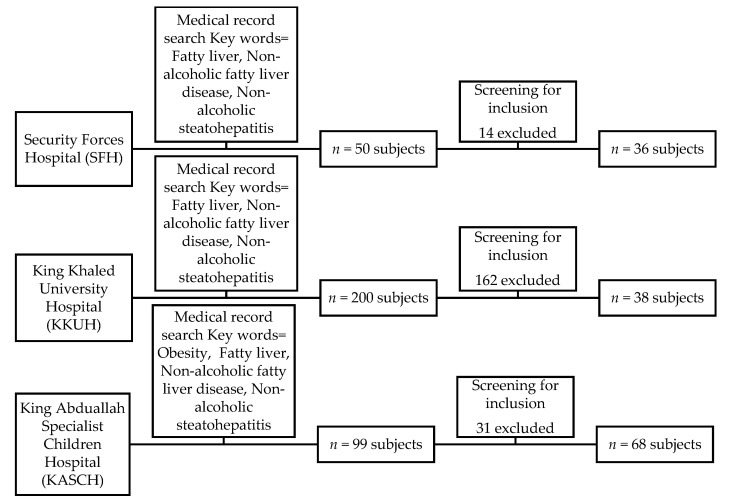
Flow chart of the study subjects’ recruitment process.

This study included 142 subjects (52.82% males). The subject’s age at the first diagnosis was categorized as 2 to 8 years (21.83%), 9 to 12 years (31.69%), 13 to 15 years (21.83%), and 16 to 18 years (24.65%). Figure 1 shows a flow chart depicting the recruitment of the subjects from each hospital based on the defined inclusion and exclusion criteria.

The subjects were divided into four groups as follows: group I, obese subjects (*n* = 37, 26.1%); group II, obese subjects with NAFLD (*n* = 74, 52.1%); group III, obese subjects with MetS (*n* = 10, 7%); and group IV, obese subjects with MetS and NAFLD (*n* = 21, 14.8%).

After reviewing patient records, the NAFLD diagnosis method and radiology report were recorded for groups II and IV. Ultrasound was the most commonly used radiological modality to diagnose NAFLD for both groups II and IV (*n* = 71, 95.9%; *n* = 20, 95.2%), respectively), followed by liver biopsy in group II (*n* = 3, 4.1%) and CT, with only one case of NAFLD diagnosed by CT (4.8%). The stages of NAFLD were mild in both groups. Table 2 shows the breakdown of the NAFLD diagnostic method and stages of NAFLD observed among the groups.

A baseline assessment of disease parameters was performed for all subjects. The baseline means and standard deviation (SD) values were computed among the four groups. BMI differed significantly during the three assessment periods among all groups (*p* < 0.05), with the mean BMI increasing after both 6 and 12 months from baseline. In group II, the mean ALP level displayed significant changes with an increase after 6 months followed by a decrease after 12 months; however, the latter value was still significantly higher than baseline (*p* = 0.034). In group IV, the mean LDL level differed significantly during the three assessment periods (*p* = 0.05), with decreases both 6 and 12 months after baseline. The other groups showed no significant differences in these disease parameters (Table 3).

The radiologist reports and liver biopsy histology reports of the study subjects diagnosed with NAFLD were reviewed, and the diagnosis criteria were applied to determine the stage of NAFLD. The NAFLD stages at baseline were distributed as follows: mild (96%), moderate (1%), and advanced (3%) among the study’s population. After 6 months the percentage of advanced-stage cases increased to 18% while the percentage of mild cases decreased to 82%. Twelve months after baseline, 11% of the study population was classified with advanced stage NAFLD and 89% had mild NAFLD. 

### 3.2. Audit of the NCP Documentation

Fifty-eight dietitian documentations were collected and audited from the three tertiary care hospitals. There is a clear difference in the quality of documentation across sites, with Hospital A demonstrating a higher quality on the audit (Table 4).

The impact of dietitian documentation on the progression of the disease parameters is plotted in Table 5. The results indicated that there was no significant effect of dietitian documentation on the disease parameters among the two groups.

The linear mixed-effects model (MIXED) procedure was conducted with changes in nutritional outcomes as outcome variables and dietetic notes categories as the predictor, and the hospital is nested as a random factor. There was no significant effect of the level of dietitian documentation on the nutritional outcomes, however, it was found that there was a significant effect of the hospital on the audit scores when testing the effect between variables in SBP (*p* < 0.001), TG (*p* = 0.005), FBG (*p* < 0.001), and bilirubin (*p* < 0.001) (Table 6).

### 3.3. Correlation between Dietitian Documentation Audit Scores and Achievement of the Nutritional Goals

Spearman’s correlation test showed a significant negative correlation between BMI and audit scores between 6 and 12 months (*r* = −0.761, *p* = 0.007), as well as a negative correlation of ALP with audit scores 12 months after baseline (*r* = −0.790, *p* = 0.02), in the group II. The other correlations between the total scores of the NCP audit and disease progression are shown in Table 7.

## 4. Discussion

There are limited available studies in Saudi Arabia that aimed to evaluate dietitian documentation in obese pediatric patients diagnosed with NAFLD, MetS, or in patients in general. Therefore, this study had two aims. The first aim was to evaluate NCP documentation in obese patients’ medical records in Saudi tertiary care hospitals in Riyadh who were diagnosed with MetS and/or NAFLD. The second aim was to evaluate the impact of dietitian documentation on the achievement of nutritional goals among the study subjects.

The first main finding of the study was related to the level of dietitian documentation among the three tertiary care hospitals. More than half of the dietitian documentations had scores of levels B and C (36.2% and 17.2%, respectively), and two of the three major tertiary hospitals in Riyadh had scores of levels B and C in the dietitian documentation audit. In Hospital B, most dietitian documentations received level B and C scores (37.5%); moreover, this hospital had the fewest documentations of the NCP with only eight dietitian notes found in the subjects’ medical records. Conversely, Hospital A had the highest audit score for dietitian notes with 69.4% of the notes scoring level A; this hospital had the highest documentation of the NCP with 36 documentations (69.4%). Regarding the quality of documentation, the best hospital was hospital A this is might be attributed to being a university hospital with regular audits training workshops in addition to having an updated electronic filing system that mandates NCP documentation with good quality. No significant differences were reported in the progression of disease parameters between the subjects with reported dietitian documentation and the subjects with no dietitian documentation.

The second main focus of this study was to explore the correlation between the total dietitian documentation scores for the NCP and the achievement of nutritional goals. Obese subjects diagnosed with NAFLD had a significant negative correlation between the audit score and change in BMI from 6 to 12 months (*r* = −0.761, *p* = 0.007). In the same group of subjects, ALP was significantly negatively correlated with audit score (*r* = −0.790, *p* = 0.02). There was also a significant negative correlation between ALP and audit score (*r* = −0.819, *p* = 0.013).

The results indicated that there is a severe lack of dietitian documentation in medical records with more than half of the documentations classified in the medium and low levels and two of the major tertiary hospitals receiving audit documentation scores of primarily levels B and C. This result was not consistent with the previous research team that developed the same tool and found that 98% of the dietitian notes received scores of level B (61%) and level C (37%) [7].

The lack of documentation can be attributed to several factors. For example, one qualitative study investigated the views of nurses regarding the cause of suboptimal documentation in patient’s charts [25]. The nurses in that study reported that inflexible charting systems, insufficient time, lack of confidence, and difficulty writing in medical charts were all factors believed to contribute to insufficient documentation [25]. Whereas the previous study focused on nursing staff, Alkhaldy et al. explored the experience of 56 Saudi dietitians in implementing the NCP [26]. The results of their study indicated that although 98% of dietitians have knowledge about the NCP, only 27% had received training in implementing the NCP in clinical practice. The dietitians reported that they were not implementing the NCP in hospitals for the following reasons: insufficient dietitians, lack of practical experience, and conflict raised from hospitals’ nutrition care systems.

Several factors play a critical role in implementing the NCP in dietitian practice. A study by Memmer reported their experience in implementing the NCP in a dialysis unit [27]. They reported that the NCP should be tailored to the setting it will be used in for easier implementation. They also found that allowing enough time to refine the implementation of the new documentation style and to adjust to the change was important [27].

Only one previous study has been conducted in Saudi Arabia, specifically in Jeddah’s hospitals, that explores the experiences of dietitians in implementing the NCP in their practices. In this cross-sectional study, 56 dietitians were recruited from six principal hospitals to answer a questionnaire about dietitian characteristics, clinical nutrition care in the hospital, perception, and opinions of dietitians toward the NCP, and the status of NCP implementation [26]. The study concluded that 98% of the dietitians were aware of the NCP; however, only 27% of the dietitians had received official training in implementing the NCP in clinical practice. Moreover, 27% of the dietitians reported that NCP documentation is challenging [26]. This study is supported by previous research that aimed to implement and monitor the NCP in dietitians’ practice, and it concluded that orienting dietitians to the NCP and monitoring their practice via chart audits are critical [28].

Although Hospital B recently implemented the NCP and ADIME in their systems, there is no documented implementation strategy utilized for implementing and monitoring this practice. The low audit scores in Hospital B and Hospital C suggest that the NCP implementation strategy must be revised or tailored to fit the settings of both hospitals. Another interpretation of the low audit scores might be that having so few dietitian documentations in the subject files in Hospital B and Hospital C compared with Hospital A contributed to their low scores.

There are several approaches that can be taken to successfully implement the NCP in clinical practice. Wills-Gallagher et al. recommend that standardization of the template and the system for NCP documentation in clinical nutrition practice should be planned and tailored according to the setting and the user of the template [29]. Their study aimed to describe how to document a patient care plan utilizing a standardized template designed based on the NCP and NCPT; a committee consisting of different healthcare professionals added input in the design of the template. After finalizing the template, they piloted it among the staff and observed increased consistency in documentation and the increased use of nutrition diagnosis codes in the patient medical records [29].

Another key finding of this study was the positive impact of high dietitian documentation scores on the improvement of some nutritional goals and outcomes. The link between dietitian documentation and disease outcomes has been investigated in several studies in different healthcare professions. In a recent systematic review conducted by the Cochrane Library, Hardiker et al. assessed the nursing records system on overall practice and patient outcomes. The systematic review included nine trials and found that although the plans documented in the nursing records had been achieved, there were no significant improvements in disease outcomes. However, they recommended that further research should be conducted on the specifically required information that must be included in the medical record [30]. Although no studies have investigated the effect of dietitian documentation on disease outcomes in patients with NAFLD, several studies have investigated the effect of early identification and documentation of malnutrition on patient outcomes.

In a retrospective study by Weddle et al., 75% of the 172 subjects received NCPs from dietitians, and the odds of achieving the recommended energy intake goal were four times greater when the documented recommendations of the dietitians were followed [31]. The study concluded that there is a positive association between dietitian care plans and the achievement of enteral nutrition outcomes of care [31]. In a prospective study by Nygaard et al., the documentation of nutritional therapy of patients in surgical gastrointestinal and orthopedic wards was assessed [32]. Of the 244 patients included in the study, 94 were diagnosed with malnutrition. The study found that some patients were not given adequate intervention, and patient monitoring was not optimum [32].

Similar results were found in a more recent study that aimed to describe the early documentation of a caloric requirement in critically ill children and the effect it has on daily energy intake. The study concluded that documentation of the caloric intake in the child’s medical record was positively associated with higher energy intake (*p* < 0.001) [33]. Another angle of the importance of dietitian documentation and its effect on patient outcomes is that it affects communication among the healthcare team, which in turn enhances patient management and patient outcomes.

Funk and Aytin conducted a retrospective review of the medical records of 234 patients diagnosed with malnutrition. They found that effective identification and documentation of patients, accurate coding of patients, and accurate malnutrition coding led to enhanced interdisciplinary communication [34]. To our knowledge, this study was the first to assess the level of documentation and the effect of the level of documentation on patient outcomes in Saudi Arabia. It was also the first to utilize the NCP audit tool in Saudi dietitian documentation in three tertiary care hospitals.

This study has several limitations. Given the rarity of NAFLD and MetS in pediatric patients, the sample size was small, which makes generalizing the results of the study difficult. Another limitation resulting from the small sample size was that some of the correlations were non-significant, potentially due to the small sample. Furthermore, the transition of medical records from paper-based records to electronic systems led to difficulty in tracking subjects before 2015 in Hospital A and Hospital B for subjects with inactive medical records or subjects who stopped visiting the hospital. The Hospital C electronic system still relies on paper-based scans of the healthcare providers’ documentation including physicians’ and dietitians’ notes, which created difficulty in tracking the subjects’ diagnosis and status.

## 5. Conclusions

This study indicated that there was a severe lack of dietitian documentation among the study population, which is critical for nutritional interventions because obesity, NAFLD, and MetS are first tackled through nutritional interventions. One explanation for this result is the transition from paper-based documentation systems to electronic documentation systems. Further research should be conducted to investigate the reasons for documentation deficiency among dietitians in tertiary care hospitals. The level of dietitian documentation was high in only one hospital whereas the other hospitals primarily had medium and low dietitian documentation scores. The audit tool utilized for assessing the level of dietitian documentation was designed utilizing the NCP, which was recently implemented in Saudi hospitals. The low documentation scores can also be attributed to the transition from the SOAP format to ADIME. However, further research should be performed to investigate the level of dietitian documentation among a larger population and in different clinical settings. Additionally, further research should be conducted to investigate current strategies utilized to ensure proper implementation of NCPs in clinical practice. This study also found that there is an association between the quality of dietitian documentation and patient nutritional outcomes. Although the literature supports the association between dietitian documentation and the improvement of patient outcomes in malnourished patients, no previous research has focused on the current study population, i.e., pediatric patients with obesity, NAFLD, and MetS. Further research should be performed to investigate the association between the quality of dietitian documentation and nutritional outcomes in a larger population.

## Figures and Tables

**Table 1:** Diet-NCP-Audit instrument. This table was adapted with permission from the author and publisher * [7].

Are the Following Statements Consistent with the Medical Record Reviewed? Answer in Accordance with the Scoring Scale, and with Support of the Associated Manual. Scoring Items 1–12: Yes = 2 p, Partly = 1 p, No = 0 p; Scoring items 13–14: Yes = 1 p, Partly = 0.5 p, No = 0 p				
Item No.	Question	Score		
1.	One or more nutrition problems have been identified and prioritized	0	1	2
2.	Possible etiology related to one or more nutrition problems is documented	0	1	2
3.	The documentation refers to signs and/or symptoms related to one or more nutrition problems	0	1	2
4.	The documentation includes relationship between problem, etiology and signs/symptoms	0	1	2
5.	The documentation includes a nutrition prescription	0	1	2
6.	The documentation includes taken or planned interventions, alternatively a comment explaining why interventions were not taken	0	1	2
7.	The documentation includes evidence for the choice of taken or planned interventions, alternatively the decision of not taking any interventions	0	1	2
8.	The documentation includes one or more goals for the intervention	0	1	2
9.	The documentation includes information of whether a follow-up appointment is planned, alternatively whether the patient is discharged	0	1	2
10.	The documentation includes a plan for how to perform the monitoring and evaluation, alternatively an explanation of why no monitoring and evaluation was planned	0	1	2
11.	The structure of the note follows the assessment-diagnosis-intervention-monitoring/evaluation format	0	1	2
12.	The language in the record is clear and may not lead to misunderstandings	0	1	2
13.	All included information is relevant for understanding the patient’s nutritional status, problem, and situation	0	0.5	1
14.	All relevant information included in the assessment part gets the response in the intervention part	0	1.5	1
Total score (max 26)		Final Score: …………….		

* The Diet-NCP-Audit instrument. Earlier published in Scandinavian Journal of Caring Science.

**Table 2:** NAFLD diagnostic method and baseline stage in obese subjects diagnosed with NAFLD.

Disease Group	NAFLD Diagnosis Method			Stage of NAFLD		
	Ultrasound *N* (%)	Liver Biopsy *N* (%)	CT *N* (%)	Mild *N* (%)	Moderate *N* (%)	Advance *N* (%)
Group II Obese with non-alcoholic fatty liver disease	71 (95.9%)	3 (4.1%)	0 (0.0%)	69 (94.5%)	1 (1.4%)	3 (4.1%)
Group IV Obese with metabolic syndrome and non-alcoholic fatty liver disease	20 (95.2%)	0 (0.0%)	1 (4.8%)	21 (100%)	0 (0.0%)	0 (0.0%)

NAFLD; non-alcoholic fatty liver disease, CT; computerized tomography.

**Table 3:** Baseline characteristics of the subjects among the four groups.

Disease Parameters	Group I	Group II	Group III	Group IV	*p*-Value
	(*n* = 37)	(*n* = 74)	(*n* = 10)	(*n* = 21)	
	Mean ± SD	Mean ± SD	Mean ± SD	Mean ± SD	
Body mass index (kg/m^2^)	30.47 ± 6.49	34.46 ± 8.03	31.55 ± 4.61	34.83 ± 6.68	0.039 **
Systolic blood pressure (mmHg)	118.27 ± 8.44	121.58 ± 14.99	116.10 ± 13.28	129.71 ± 16.43	0.012 **
Diastolic blood pressure (mmHg)	66.49 ± 8.97	69.57 ± 9.8	67.40 ± 9.29	72.05 ± 8.80	0.152
Triglycerides (mmol/L)	0.98 ± 0.44	1.33 ± 0.85	1.08 ± 0.36	1.56 ± 0.69	0.50
Total cholesterol (mmol/L)	4.34 ± 0.68	4.19 ± 0.86	4.32 ± 1.08	4.70 ± 0.97	0.173
High-density lipoprotein (mmol/L)	1.13 ± 0.22	1.31 ± 0.88	1.00 ± 0.23	1.10 ± 0.40	0.360
Low-density lipoprotein (mmol/L)	2.81 ± 0.64	2.54 ± 0.71	3.11 ± 1.12	2.91 ± 0.82	0.68
Insulin (mIU/L)	18.71 ± 15.91	25.71 ± 13.37	N/A *	33.11 ± 18.32	0.308
Fasting blood glucose (mmol/L)	4.90 ± 0.43	5.69 ± 3.47	5.18 ± 0.58	9.26 ± 6.51	0.006 **
Alanine aminotransferase (U/L)	24.97 ± 14.39	46.84 ± 59.15	30.50 ± 17.59	52.74 ± 32.75	0.110
Aspartate aminotransferase (U/L)	24.93 ± 7.86	36.46 ± 38.52	30.83 ± 11.58	32.46 ± 16.18	0.373
Alkaline phosphatase (U/L)	250.34 ± 66.32	182.61 ± 87.07	269.14 ± 28.29	178.55 ± 88.25	<0.001 **
Gamma-glutamyltransferase (U/L)	N/A *	42.91 ± 109.77	N/A *	38.05 ± 27.57	0.460
Bilirubin (umol/L)	6.69 ± 3.49	13.31 ± 41.00	6.97 ± 2.27	7.84 ± 3.36	0.741
Prothrombin time (seconds)	10.84 ± 0.48	11.97 ± 2.19	11.50 ± 0.57	13.17 ± 4.35	0.358
C-reactive protein (mg/L)	27.00 ± 35.36	13.87 ± 31.47	N/A *	13.51 ± 14.19	0.844

* N/A = Not available due to missing data. ** Significant difference. One-way ANOVA was utilized for the analysis.

**Table 4:** Comparison of the audit results of the NCP documentation in three tertiary care hospitals in Riyadh.

Hospital	Audit Results			*p*-Value
	Level A (Total Score = 20–26)	Level B (Total Score = 13–19)	Level C (Total Score = 0–13)	
	*n* (%)	*n* (%)	*n* (%)	
Hospital A (*n* = 36)	25 (69.4%)	9 (25%)	2 (5.6%)	<0.001 *
Hospital B (*n* = 8)	2 (25%)	3 (37.5%)	3 (37.5%)	
Hospital C (*n* = 14)	0 (0%)	9 (64.3%)	5 (35.7%)	
Total (*n* = 58)	27 (46.6%)	21 (36.2%)	10 (17.2%)	

* Significant difference (*p* < 0.05). One-way ANOVA was utilized for the analysis.

**Table 5:** The disease parameters in subjects with and without reported dietitian documentation.

Disease Parameters	Reported Dietitian Documentation	No Reported Dietitian Documentation	*p*-Value
	*n* = 58	*n* = 84	
	Mean ± SD	Mean ± SD	
Change in body mass index (kg/m^2^) *	−1.25 ± 2.97	−1.07 ± 1.78	0.724
Change in systolic blood pressure mmHg	−1.62 ± 14.82	−1.73 ± 13.96	0.971
Change in diastolic blood pressure mmHg	−0.86 ± 10.88	−1.79 ± 10.34	0.677
Change in triglycerides (mmol/L)	−0.03 ± 0.46	0.01 ± 0.0.77	0.854
Change in total cholesterol (mmol/L)	0.04 ± 0.70	0.31 ± 0.99	0.275
Change in high-density lipoprotein (mmol/L)	0.03 ± 0.24	0.34 ± 1.30	0.261
Change in low-density lipoprotein (mmol/L)	0.04 ± 0.67	0.09 ± 0.47	0.734
Change in fasting blood glucose (mmol/L)	0.59 ± 3.43	0.34 ± 3.28	0.869
Change in alanine aminotransferase (U/L)	−2.87 ± 40.76	8.00 ± 24.39	0.261
Change in aspartate aminotransferase (U/L)	−1.38 ± 23.04	4.87 ± 21.81	0.346
Change in alkaline phosphatase (U/L)	2.35 ± 54.77	−8.29 ± 70.92	0.569
Change in gamma-glutamyl transferase (U/L)	−7.08 ± 26.06	5.25 ± 16.14	0.135
Change in bilirubin (umol/L)	−6.52 ± 30.93	−15.76 ± 57.46	0.499
Change in prothrombin time (seconds)	−1.80 ± 2.07	−1.03 ± 2.01	0.574

* The changes were calculated as follows: change from baseline to 12 months = 1st assessment–3rd assessment. The independent sample *t*-test was utilized for the analysis.

**Table 6:** Effect of dietitian documentation level on disease parameters in subjects with reported dietitian documentation among the entire study population.

Disease Parameters	Level A (Total Score = 20–26)	Level B (Total Score = 13–19)	Level C (Total Score = 0–13)	*p*-Value
	*n* = 27	*n* = 21	*n* = 10	
	Mean ± SD	Mean ± SD	Mean ± SD	
Change in body mass index (kg/m^2^) *	−1.56 ± 3.54	−0.39 ± 1.53	−2.10 ± 3.18	0.579
Change in systolic blood pressure (mmHg)	−5.43 ± 14.29	4.23 ± 12.51	−1.13 ± 18.11	0.453
Change in diastolic blood pressure (mmHg)	−5.48 ± 8.11	3.54 ± 9.59	4.13 ± 14.83	0.218
Change in triglycerides (mmol/L)	−0.08 ± 0.54	−0.04 ± 0.47	0.08 ± 0.23	0.542
Change in total cholesterol (mmol/L)	0.06 ± 0.69	0.44 ± 0.60	−0.58 ± 0.42	0.119
Change in high-density lipoprotein (mmol/L)	0.04 ± 0.29	0.09 ± 0.21	−0.06 ± 0.07	0.215
Change in low-density lipoprotein (mmol/L)	0.06 ± 0.62	0.41 ± 0.67	−0.55 ± 0.38	0.103
Change in fasting blood glucose (mmol/L)	−0.03 ± 0.41	−0.79 ± 1.67	6.50 ± 8.34	0.777
Change in alanine aminotransferase (U/L)	−2.15 ± 10.74	1.79 ± 23.38	−22.33 ± 121.16	0.364
Change in aspartate aminotransferase (U/L)	−2.00 ± 17.86	1.17 ± 8.94	−8.67 ± 64.44	0.452
Change in alkaline phosphatase (U/L)	−11.33 ± 53.24	−0.09 ± 38.49	52.33 ± 98.08	0.182
Change in bilirubin (umol/L)	−0.13 ± 2.51	−0.12 ± 1.70	−49.20 ± 85.68	0.420

* The changes were calculated as follows: change from baseline to 12 months = 1st assessment–3rd assessment.

**Table 7:** Correlation of audit scores with disease progression among the four groups.

Disease Parameters	Change from Baseline to 6 Months *		Change from 6 Months to 12 Months *		Change from Baseline to 12 Months *	
	*r*	*p*-Value	*r*	*p*-Value	*r*	*p*-Value
Group I						
Change in body mass index	−0.275	0.241	−0.445	0.084	−0.315	0.188
Group II						
Change in body mass index	−0.298	0.322	−0.761 **	0.007	−0.326	0.301
Change in alanine aminotransferase	0.299	0.471	0.632	0.368	0.006	0.989
Change in aspartate aminotransferase	0.427	0.292	−0.800	0.200	0.291	0.485
Change in alkaline phosphatase	−0.275	0.509	0.400	0.600	−0.790 **	0.02
Change in gamma-glutamyl transferase	−0.090	0.848	NA ***	NA ***	−0.116	0.827
Change in bilirubin	−0.299	0.471	NA ***	NA ***	0.240	0.568
Group III						
Change in body mass index	0.019	0.968	0.316	0.684	0.316	0.684
Change in systolic blood pressure	0.561	0.190	−0.821	0.089	0.526	0.362
Change in diastolic blood pressure	0.661	0.106	−0.821	0.089	−0.205	0.741
Change in total cholesterol	NA ***	NA ***	NA ***	NA ***	0.500	0.667
Change in high-density lipoprotein	NA ***	NA ***	NA ***	NA ***	0.500	0.667
Change in low-density lipoprotein	NA ***	NA ***	NA ***	NA ***	0.500	0.667
Group IV						
Change in body mass index	−0.410	0.273	0.180	0.699	0.054	0.908
Change in systolic blood pressure	0.030	0.933	−0.631	0.129	−0.627	0.132
Change in diastolic blood pressure	0.027	0.940	0.711	0.074	−0.100	0.831
Change in triglycerides	−0.616	0.269	NA ***	NA ***	−0.559	0.192
Change in total cholesterol	−0.872	0.054	−0.866	0.333	0.577	0.175
Change in high-density lipoprotein	−0.616	0.269	0.866	0.333	0.667	0.102
Change in low-density lipoprotein	−0.718	0.172	−0.866	0.333	0.739	0.58
Change in fasting blood glucose	0.500	0.667	NA ***	NA ***	NA ***	NA ***
Change in alanine aminotransferase	−0.500	0.667	NA ***	NA ***	−0.414	0.355
Change in aspartate aminotransferase	−0.500	0.667	−0.500	0.667	−0.414	0.355
Change in alkaline phosphatase	−0.500	0.667	−0.500	0.667	0.036	0.939
Change in gamma-glutamyl transferase	0.500	0.667	−0.500	0.667	−0.029	0.957
Change in bilirubin	NA ***	NA ***	−0.500	0.667	−0.234	0.613

* The changes were calculated as follows: change from baseline to 6 months = 1st assessment–2nd assessment, change from 6 to 12 months = 2nd assessment–3rd assessment, change from baseline to 12 months = 1st assessment–3rd assessment. ** Correlation is significant (*p* < 0.05). *** Correlation and/or *p*-value cannot be calculated due to missing cases. Spearman’s correlation test was utilized for the analysis.

## Data Availability

Original data supporting these results are available on request from the corresponding author for reasonable purposes.
